# Reliable Ultra Trace Analysis of Cd, U and Zn Concentrations in Greenland Snow and Ice by Using Ultraclean Methods for Contamination Control

**DOI:** 10.3390/molecules25112519

**Published:** 2020-05-28

**Authors:** Changhee Han, Heejin Hwang, Jung-Ho Kang, Sang-Bum Hong, Yeongcheol Han, Khanghyun Lee, Soon Do Hur, Sungmin Hong

**Affiliations:** 1 Division of Polar Paleoenvironment, Korea Polar Research Institute, 26 Songdomirae-ro, Yeonsu-gu, Incheon 21990, Korea; hch@kopri.re.kr (C.H.); heejin@kopri.re.kr (H.H.); jhkang@kopri.re.kr (J.-H.K.); hong909@kopri.re.kr (S.-B.H.); yhan@kopri.re.kr (Y.H.); leekh@kopri.re.kr (K.L.); sdhur@kopri.re.kr (S.D.H.); 2 Department of Ocean Sciences, Inha University, 100 Inha-ro, Michuhol-gu, Incheon 22212, Korea

**Keywords:** ultraclean procedure, ultralow trace elements, contamination, Greenland snow and ice, seasonal variations, anthropogenic inputs

## Abstract

This study presents ultraclean procedures used in the challenging task of determining trace elements at or below the pg/g concentration level encountered in Greenland snow and ice. In order to validate these ultraclean procedures, recent snowfall and Holocene ice from northwest Greenland were analyzed for Cd, U, and Zn concentrations. The total procedural blanks brought through the entire measurement procedure proved to be negligible, compared to trace element concentrations, measured in snow and ice samples. This validates the overall practicality of the proposed ultraclean procedures, thereby ensuring the reliable measurements of ultra-trace analysis. A comparison between our study and published data shows that improper procedures employed throughout all stages, from field sampling to analysis to elevate the concentrations by several orders of magnitude, relative to the reliable concentration ranges. The risk of contamination exposure for selected trace elements appears to increase in the order of U < As ≤ Pb < Cd < Zn. Reliable measurements of Cd, U, and Zn concentrations in snow and ice allowed us to interpret the data in terms of seasonal variations in the inputs of crustal and anthropogenic sources to Greenland ice sheet.

## 1. Introduction

Since a pioneering investigation by C. Patterson and his co-workers [1], successive Greenland snow and ice layers have proved to be valuable archives for characterizing and understanding human-induced changes in the large-scale atmospheric cycles of toxic trace elements in the northern hemisphere, over time scales of several tens of years to millennia [2,3,4,5,6,7,8,9]. Despite the fact that such data are of great concern to assess to what extent they have been altered by human perturbation, comprehensive data remain very limited, mainly because trace element concentrations for measurement in Greenland snow and ice are extremely low at low and sub-pg/g (1 pg/g = 10^−12^ g/g) levels. The determination of such ultralow trace element concentrations has proved to be an analytical challenge, in terms of the difficulties encountered, while trying to overcome the risk of sample contamination during all stages of field sampling, storage, handling, and analysis [10,11,12,13,14,15,16,17,18]. Indeed, previous Greenland snow and ice data (e.g., Cd, Zn, and Hg) published in the 1970s [19,20,21,22] were later revealed to be erroneously high, by as much as several orders of magnitude when significant contamination problems at the pg/g level were fully realized and stringent strategies to minimize and control the risk of contamination, from field sampling to analysis, were subsequently developed [3,10,23,24,25]. Obviously, erroneous data ultimately lead to quantitatively and qualitatively incorrect interpretation of the data.

Although such contamination has been known for ~40 years as a critical factor affecting the reliable measurement of ultralow trace elements in Greenland snow and ice [23,24], several laboratories still face challenges in reducing contamination. It is difficult to properly evaluate the nature, extent and magnitude of contamination at individual laboratories because of the large and complex contamination source and because contaminants can infiltrate at all stages of the entire procedures from field sampling and laboratory analysis. Furthermore, the development and implementation of ultraclean methods to successfully avoid contamination problems require long-term experience and practice in the workplace. In this context, an effective and indirect approach to ensure the cleanness of all the procedures requires an examination of whether the data obtained are within the ranges of previously published reliable concentrations of trace elements investigated [3].

This study aims; (1) to present and validate all our practical ultraclean procedures employed in determining trace elements in Greenland snow and ice; (2) to analyze cadmium (Cd) and uranium (U) at extremely low concentrations down to the sub-pg/g and zinc (Zn), an element that is highly prone to contamination, in snow and Holocene ice using these ultraclean procedures; (3) to compare their concentrations (including As and Pb previously determined in the same samples) with previous data to assess to what extent these elements are easily exposed to contamination; and finally, (4) to investigate short-term (intra- and inter-annual) variations and the relative magnitude of natural and anthropogenic inputs of Cd, U, and Zn in recent Greenland snow layers dated from 2003 to 2009.

## 2. Results and Discussion

### 2.1. Total Procedural Blank Contribution

The estimated total procedural blank (TPB) contributions of the analyzed trace elements from the snow sampling and artificial ice cores (AICs) decontamination procedures are shown in Table 1. The results show that no detectable contributions were introduced due to the complete procedure from field snow sampling to analysis. The TPB determined for the AIC decontamination procedure was 0.108 pg/g for As, 0.016 pg/g for Cd, 0.061 pg/g for Pb, 0.009 pg/g for U, and 0.632 pg/g for Zn, which are equivalent to ~2–8% of the lowest concentrations of a given element measured in the Holocene ice samples from the North Greenland Eemian Ice Drilling (NEEM) deep ice core (Table 2). This indicates that our ultraclean procedures attain consistently high quality contamination control, required for reliable measurements of trace elements at the extremely low concentration levels encountered in Greenland snow and ice. Blank corrections were not applied to the data obtained from this study because of the negligible amount of contaminants introduced during our complete procedure.

### 2.2. Cd, U and Zn Concentrations in the NEEM Snow and Ice Samples

**Figure 1:**
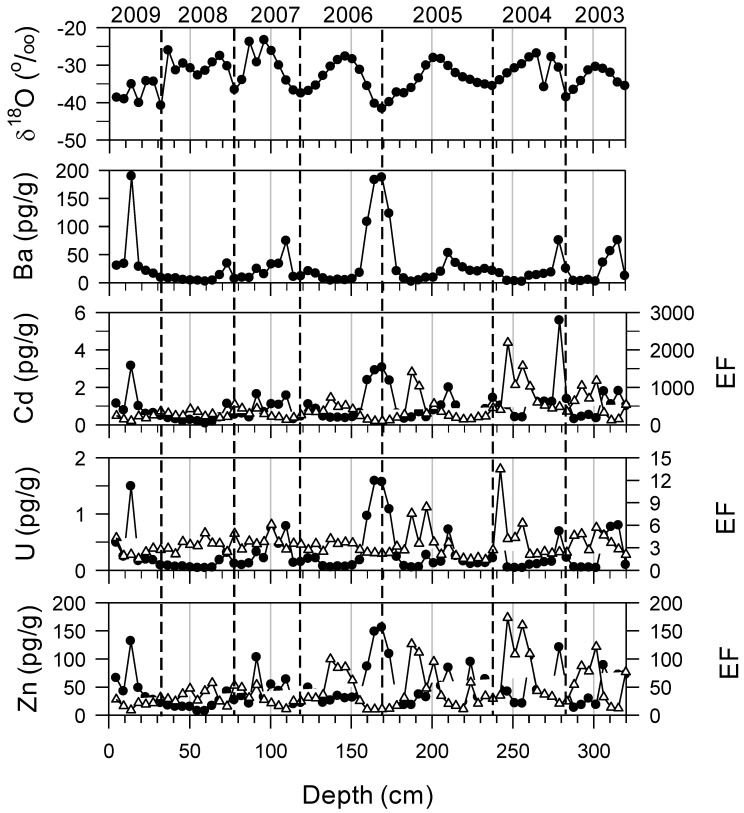
Depth profiles of δ^18^O, Ba, Cd, U, and Zn concentrations (solid circles) and crustal enrichment factors (EF) (open triangles) determined in the NEEM snow pit samples in Greenland. Vertical dashed lines represent the minima of δ^18^O in winter during the period 2003–2009.

The concentrations of Cd, U and Zn, measured in a continuous series of 70 samples from the NEEM snow pit, are shown in Figure 1 as a function of depth and age. All data are listed in Table S1 of the supplementary material. Figure 1 shows that the concentration levels are highly variable with depth. Such a strong variability in concentrations is associated with intra-annual (seasonal) variations in the transport and subsequent deposition processes of individual elements to Greenland snow as previously found elsewhere in Greenland [26,27,29,31,33,36,37]. Table 2 summarizes the statistics for the observed concentrations. The amplitude of the variations differs from one element to another, with the ratios between maximum and minimum concentrations of about 80 for Cd, 40 for U, and 22 for Zn (Table 2). The highest ratio between maximum and minimum concentrations for Cd is influenced by the single very high value of 5.57 pg/g (Figure 1 and Table S1). The measured concentrations (mean values ± SDs) are 0.97 ± 0.89 pg/g for Cd, 0.29 ± 0.37 pg/g for U, and 46.4 ± 33.5 pg/g for Zn, confirming that the present-day Greenland snow is very pure for these elements with the concentration levels varying from tens of pg/g down to sub-pg/g.

The concentrations of Cd, U, and Zn measured in the NEEM deep ice core dated back to ~8266–9166 years before present (BP, where present is defined as 1950), as Holocene natural background samples, are 0.20–0.40 pg/g (mean value: 0.32 pg/g) for Cd, 0.21–0.50 pg/g (mean value: 0.34 pg/g) for U and 27.1–42.2 pg/g (mean value: 33.8 pg/g) for Zn (Table 2). The abnormally high concentrations observed for Cd and Zn in the 1215.50–1215.70 m section (~8056 years BP) (Table 2) are one or two orders of magnitude larger than those in the other Holocene ice samples, undoubtedly due to the infiltration of enormous external contamination into the very center of the core section (see Section 2.4). We excluded the concentrations of individual elements measured in this ice section in averaging the Holocene natural concentrations. Besides U, for which data are not available, the only reliable data ever published on Holocene natural concentration values of Cd and Zn in Greenland ice were obtained by analyzing three core sections, dated from 8250 to 9310 years BP, of the GRIP deep ice core drilled at Summit [35]. Despite differences in locations between the NEEM and GRIP sites (Figure S1), probably resulting in spatial variation in concentrations from site to site, Cd and Zn concentrations measured in the central parts of these core sections are in good agreement with ours (Table 2), confirming the reliability of our data.

Interestingly, the mean concentrations of Cd and Zn measured in the NEEM snow pit samples are ~3 and 1.4 times, respectively, above the NEEM Holocene natural levels, while the mean concentration of U is similar to the Holocene natural level. This suggests significant enrichments of Cd and Zn in the most recent Greenland snow, relative to the natural levels, most likely due to anthropogenic inputs, as discussed below.

### 2.3. Crustal Enrichment Factor (EF)

We calculated the crustal enrichment factor (EF) using the concentration ratio of a given element to Ba (a reference element for crustal dust) in snow [27], normalized to the same concentration ratio in the upper continental crust (UCC) [38]. If the calculated EFs are close to unity, crustal dust is a dominant source of trace elements investigated; conversely, EFs much larger than unity (>10) represent that contributions from other natural sources or anthropogenic sources are important [26]. However, the selection of a reference element (e.g., Al, Ba and Sc) and crustal compositions can influence the results of the EF calculation [39]. A supplementary approach to compensate for such uncertainties is determining the site-specific Holocene natural background levels of EFs for individual elements, and thus, to quantify the extent to which the anthropogenic contribution accounts for the atmospheric cycles of elements of interest during the investigated time period.

In Table 2, the mean (±SD) Holocene natural EFs at the NEEM site are 58 ± 27 (range: 39–88) for Cd, 2.6 ± 1.8 (range: 1.3–4.7) for U, and 12 ± 2.6 (range: 8.7–14) for Zn. These Holocene natural EF values suggest that crustal dust is the dominant source for U, while inputs from other natural sources, such as volcanic emissions and continental biogenic activity are important for Zn and especially for Cd [35,40]. This is consistent with the observation that during warm interglacial periods, crustal dust was the predominant source for U, but accounted for a small contribution to Cd and Zn in Antarctic ice [41,42]. The mean EFs (±SD) in the NEEM snow pit samples are 430 ± 376 for Cd, 3.5 ± 1.8 for U, and 44 ± 37 for Zn, which are about 7.4, 1.3, and 3.7 times, respectively, the mean Holocene natural levels (Table 2). This indicates high enrichments especially of Cd and Zn with respect to the Holocene natural levels in recent Greenland snow, most likely due to anthropogenic contribution [2,6,8,29,31].

### 2.4. Comparison with Published Data

We compared our concentration levels of Cd, U, and Zn, including As and Pb measured in the same snow pit samples [26,27], with published data available from Greenland snow layers dated from the 1980s to 2000s. Differences in geographical properties and time periods investigated occur between the data sets (Table 2 and Figure S1).

Despite the very limited data of U measured in Greenland snow, the range and mean concentration of U in our samples are lower than those given by Lai et al. [28] and Barbante et al. [29] (Table 2). Assuming that previous U data were reliable, this difference is probably partially due to the complexity of transport and deposition of U-containing aerosols to different geographical locations in Greenland [36,38,43], and/or the large inter-annual variability according to the time periods covered [8,26,31]. Alternatively, we cannot completely exclude the effect of contamination on higher U concentrations, given by Lai et al. [28] and Barbante et al. [29]. These considerations will be confirmed when long-term temporal and spatial trends in U concentrations are obtained from Greenland snow deposited between the 1960s and 2000s.

As for the other elements, unusually high concentrations of As (22 ± 29 pg/g), Cd (43 ± 50 pg/g), Pb (200 ± 690 pg/g), and Zn (4,120 ± 4,830 pg/g) were observed in the snow layers deposited in spring 2012 and 2013 and in summer 2013 and 2014, collected from 22 snow pits across central and northwest Greenland [28] (Table 2). These concentrations differ by orders of magnitude compared to ours. Furthermore, the concentrations of Cd, Pb, and Zn available from Greenland snow between the 1980s and 1990s, varying in the ranges of ~0.1–11.6 pg/g for Cd, 0.6–158 pg/g for Pb and 2–285 pg/g for Zn (Table 2), are much lower than those given by Lai et al. [28]. When comparing our data with those by Lai et al. [28], differences also exist regarding the EF values. The mean EF values by Lai et al. [28] are considerably higher (by a factor of 2.3, 13, 2.7 and 22 for As, Cd, Pb and Zn, respectively) than ours (Table 2). The authors suggested that such highly enhanced concentrations in their spring–summer samples, compared to previous data, were likely linked to the seasonality of the concentrations. As seen in Figure 1, however, our data show peak concentrations in the winter–spring snow, and much lower concentrations in the summer–autumn snow, for all elements investigated (see last section), consistent with the seasonal patterns observed in the Summit snow pit samples [29]. As the unusually high concentrations cannot be explained by spatial variations and/or seasonality in trace element concentrations within the Greenland ice sheet, it is, therefore, beyond doubt that the data by Lai et al. [28] were plagued by major contamination problems at one or several stages of field sampling, storage, handling and analysis. The data by Lai et al. [28] give a rough insight into the levels of contamination that are surprisingly high, ranging from a few tens of pg/g for As and Cd to a few ng/g for Zn, which obscure the meaning of most data of these elements in Greenland snow. The comparison of our results with those by Lai et al. [28] allows us to assess the risk of contamination exposure for selected trace elements. In Table 2, the mean concentrations by Lai et al. [28] are higher than ours by a factor of ~8.4 for As, 44 for Cd, 9.3 for Pb, 2.4 for U, and 89 for Zn, indicating that the risk of contamination exposure increases in the order of U < As ≤ Pb < Cd < Zn.

**Figure 2:**
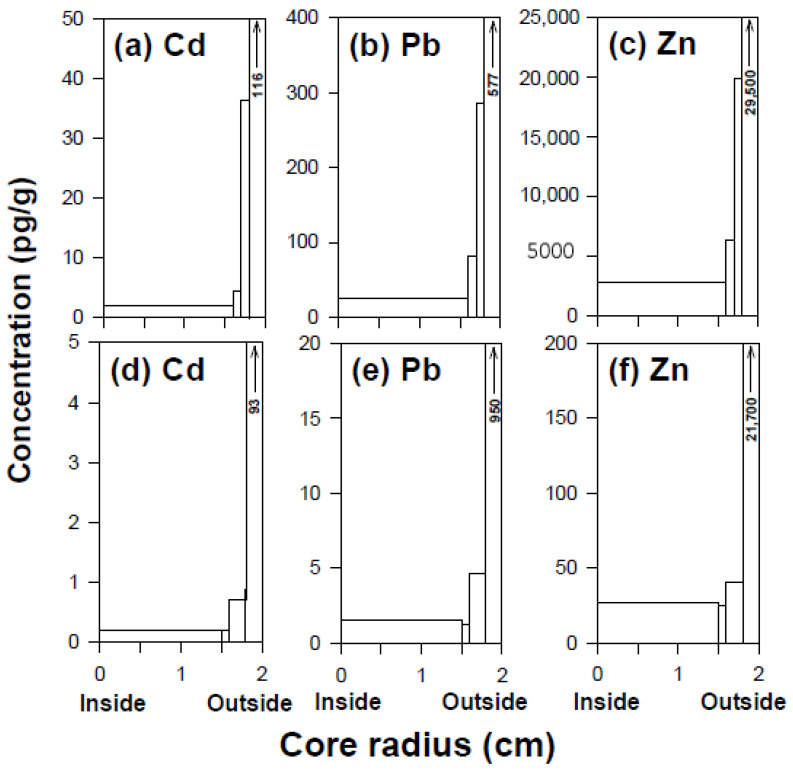
Examples of changes in Pb and Zn concentrations as a function of radius in two sections. (**a**)–(**c**) 1215.50–1215.70 m (8056 years BP); (**d**)–(**f**) 1275.45–1275.65 m (8840 years BP).

These discrepancies highlight the importance of well-established ultraclean contamination control procedures from field sampling to laboratory analysis, coupled with adequate sampling and experimental design, in order to ensure the reliability of ultra-trace analysis. In addition, TPB within reasonable levels compared to ultralow concentration levels in ultrapure Greenland snow and ice is of prime importance, as it guarantees the ultraclean procedures employed in the analysis. Otherwise, a systematic error will induce unusually high concentration levels of certain trace elements that are more easily exposed to contamination. Even when conducting ultra-trace analysis using all the ultraclean procedures, researchers have sometimes encountered unexpected contamination problems, particularly when analyzing deep ice core samples from the highly contaminated external environment existing during drilling operations, storage, and handling of ice cores [12,18]. As described before, unusually high concentrations of Cd, Pb and Zn compared to those in the other NEEM Holocene ice samples were measured in the 1215.50–1215.70 m section (8056 years BP) (Table 2). The outside to inside profiles of Cd, Pb and Zn show a continuously decreasing concentration trend, implying the transfer of extremely high external contamination into the very center of the core section (Figure 2a–2c), probably due to invisible fine cracks in the core section. Conversely, changes in concentrations show well-established plateau values in the central parts of the 1275.45–1275.65 m section (8840 years BP) (Figure 2d–2f). This indicates that the concentrations measured in the inner core represent the original concentrations in the ice [12,18].

### 2.5. Seasonal Variations in Concentrations and EFs

As displayed in Figure 1, the large variability of concentrations of Cd, U, and Zn reflects the strong seasonal variations. Our data generally show enhanced concentrations in the winter-spring layers (δ^18^O minima) for all elements, occurring in phase with pronounced crustal element, i.e., Ba, peaks (Figure 1) as seen for Pb and As in the same pit samples [26,27]. All the elements are very strongly correlated with Ba, with Pearson’s correlations of 0.767, 0.943 and 0.823 for Cd, U and Zn, respectively, that are significant at *p* = 0.01. This reflects that crustal dust is the major medium for the long-range transport of individual elements from the potential source regions to northwestern Greenland or that the contribution from crustal dust is very large. The latter can be seen for U, because of the largest correlation coefficient between U and Ba and the lowest mean EF value (3.4 ± 1.8) (Table 2).

**Figure 3:**
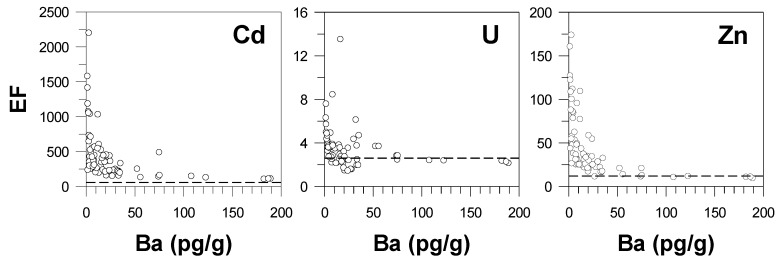
Changes in crustal enrichment factors (EF) of Cd, U, and Zn as a function of Ba concentrations. Horizontal dashed lines represent the mean Holocene natural EF values for each element (see text).

In contrast to the positive correlations of Cd, U, and Zn concentrations with respect to Ba concentrations, the EF values tend to be inversely correlated with Ba concentrations with elevated EF values when Ba concentrations are relatively low in the summer and autumn snow layers (δ^18^O maxima) (Figure 1). This feature is better illustrated in Figure 3, which shows a sharp decrease in EF values with increasing Ba concentrations. Interestingly, this relationship is also observed for U, although the crustal contribution is dominant for this element. In Figure 3, the Ba concentration exhibits an apparent critical point (~30 pg/g) beyond which the EF values approach to the Holocene natural levels. This suggests that crustal dust deposition episodes associated with Ba peaks, occurring in the winter and spring (Figure 1), are characterized by the incursion of air masses more directly from primary Asian dust source regions through the upper troposphere [36,44,45], thereby reducing the entrainment of anthropogenic pollutants. Conversely, the highly elevated EFs for all the elements above the Holocene natural levels in the summer and autumn snow layers are likely associated with the more efficient arrival of air masses bearing anthropogenic pollutants from lower latitudes [46], leading to a greater proportion of anthropogenic contribution in the corresponding snow layers [26,27].

The temporal trends of concentrations and EF values show different patterns of variation (Figure 1). However, the short six-year duration from 2003 to 2009 of our snow samples is insufficient to demonstrate any definite temporal pattern. None of the elements show any significant temporal trend in concentrations, partly due to anomalous concentration maxima, matching with Ba peaks at depths of 10–15 and 160–170 cm, during winter and spring time. Although different geographical properties, between the sites in Greenland, make it difficult to establish a well-defined temporal trend for the recent decades, comparison of our data with published data demonstrates that mean concentrations of Cd and Zn in Greenland snow have not significantly decreased between the 1980s and 2000s (Table 2). Since the mid-1970s, the emissions of trace elements, including Cd and Zn, have continuously decreased, in line with the improvements of pollution reduction technology, particularly in Europe and North America [47,48]. Conversely, the emissions of Cd and Zn in China, one of the largest emitter countries worldwide, have rapidly increased from ~100 t and ~4130 t in 1980 to ~800 t and ~20,500 t in 2010, respectively, in line with the rapid increase in energy consumption and industrial production [49,50]. Combining previous findings of a growing Chinese contribution to the anthropogenic pollutants deposited to Greenland over the past decades [8,26,27,44], we argue that the increasing Chinese contribution may have resulted in the negligible decline in Cd and Zn concentration levels in Greenland snow between the 1980s and 2000s.

As for the EF record in the snow pit, the EF peaks show an order of magnitude seasonal variability between 2003 and 2006 but, from 2007 to 2009, remained less variable at low levels with mean EF values of 273 for Cd, 3 for U, and 29 for Zn (Figure 1). This finding is most likely due to a combination of seasonal and inter-annual patterns, reflecting the complex variability of the potential sources or source regions, which influence the changing occurrence of various trace elements in Greenland snow [29,31]. The very large EF values of Cd and Zn observed in the summer and autumn snow layers between 2003 and 2006 are certainly due to strong anthropogenic inputs, as has been well documented in other snow and ice records from Greenland [2,6,8,29,31]. The low mean EFs from 2007 to 2009 exceed by a factor of 4.7 for Cd and 2.4 for Zn those of the Holocene natural levels, indicating that ~80% and ~60% of present-day Cd and Zn deposited to central Greenland is of anthropogenic origin during the corresponding period. For U, the highly enhanced EF values, reaching up to ~5 times those of the Holocene natural level (Table 2), are also likely associated with anthropogenic contribution, despite the absence of any long-term time trend in the occurrence of U in Greenland snow. Ice core studies from the Alps and Mount Everest showed that the recent increase in atmospheric concentration of U in Europe and central Asia was associated with regional U mining production [51,52]. Furthermore, the significant enrichment of U in Antarctic snow and ice over the last few decades was attributed to increased U production in South Africa and Australia [53,54]. These results suggest that human activities have substantially affected the current global atmospheric cycle of U, supporting an anthropogenic source for this element in our snow layers, which exhibited enhanced EF values above the Holocene natural level. More results are needed to confirm our observation, and to quantify the extent and impact of the anthropogenic release of U, and its long-range transport to the most remote areas in the northern hemisphere.

## 3. Experimental

Since a clean protocol for determining ultralow trace elements in polar snow and ice was provided by Hong et al. [17], further improvements in clean laboratory facilities and procedures have been made for the more routine ultra-trace analysis at the Korea Polar Research Institute (KOPRI, Incheon, Korea) [18]. We present an overview of ultraclean laboratory facilities and analytical procedures used to avoid the risk of contamination. It should be noted that ultra-trace analysis requires a high level of expertise and performance of the analysts.

### 3.1. Clean Facilities

As previously emphasized by various authors [10,11,13,15,16,17,18,23], the clean working environment is a key mandatory requirement for minimizing and controlling the risk of introducing contaminants (mostly particulates) into the samples, which arise from the laboratories. The non-laminar flow Class 1000 clean room, vertically flushed with air filtered through high efficiency particulate air (HEPA) filters (99.97% at 0.3 μm, Cambridge Filter Korea Co., Ltd., Cheongwon-gun, Korea), was designed especially for ultra-trace analysis. Due to the extensive use of concentrated acids in a cleaning laboratory labware and sampling items, all metallic components were avoided in a clean room to prevent the potential for corrosion and particle generation. More importantly, all containers and baths containing acids were placed in custom-made, polyvinyl chloride (PVC)-ducted cabinets (CA engineering Co., Ltd., Seoul, Korea) to further reduce the risk of harmful acid vapor exposure in the clean room air. The filtered air in the clean room was washed by a recirculation system through a medium filter (~85% efficiency at 0.5 μm, Cambridge Filter Korea Co., Ltd., Cheongwon-gun, Korea) and a carbon-coated filter (removing acid fumes, Cambridge Filter Korea Co., Ltd., Cheongwon-gun, Korea), and subsequently passed through HEPA filters.

The Class 10 all plastic (PVC) clean bench (1900 mm long × 900 mm wide × 2000 mm high, external dimension) and booth (3000 mm long × 2020 mm wide × 2470 mm high, external dimension) were installed in the Class 1000 clean room. The vertical laminar flow air was flushed into the bench and booth through ultra-efficiency particulate air (ULPA) filters (Cambridge Filter Korea Co., Ltd., Cheongwon-gun, Korea) (99.9995% efficiency at 0.1 μm). All procedures for the rinsing of sample bottles and containers, sample preparation, handling, and analysis that required complete contamination control were performed in these workstations. The periodic replacement of HEPA and ULPA filters and monitoring of particles, with a particle counter, were essential to maintaining air quality. The operators always wore full clean room clothing and low-density polyethylene (LDPE) gloves during operation.

### 3.2. Ultrapure Water

The purity of water is of particular importance because water is a major source of contamination in samples and regents. In our laboratory, two grades of ultrapure water were used: Millipore Milli-Q (MQW) ultrapure water (18.2 MΩ∙cm resistivity) produced by combining a Millipore RO water purifier (Model Elix-3) with a Milli-Q system (Model Milli-Q Academic, Millipore Corp., Darmstadt, Germany), fed by tap water, passed through a succession of micron-level and carbon pre-filters; and sub-boiling distilled ultrapure water (SDW) produced by sub-boiling distillation equipment with two high-purity quartz distillation units (DuoPUR, Milestone, Sorisole, Italy), using the MQW. The MQW and SDW were produced inside a Class 10 clean booth and the final output rate of the SDW was limited to ~500 mL/day.

The MQW was extensively used during the various steps of the cleaning procedures, while the SDW was used for the final rinsing of acid-cleaned plastic bottles and containers (see next section), prior to the storage of solutions of high purity, such as samples and standards. All the items used for snow sampling and decontamination procedure of ice core sections were also cleaned with the SDW before use.

Concentrations of selected trace elements were measured in the MQW and SDW, after being pre-concentrated by non-boiling evaporation and acidification to 1% using Fisher “Optima” grade ultrapure HNO_3_ [55], using ultrasensitive inductively coupled plasma sector field mass spectrometry (ICP-SFMS, Element2, Thermo Fisher Scientific, Bremen, Germany) (Table 1). The SDW was ~2–3 times cleaner for Ba, Cd and Pb than the MQW was, while U and Zn showed a comparable level of concentration between SDW and MQW. Interestingly, As shows higher concentration in the SDW, although the concentrations of As in the MQW and SDW were extremely low at the sub pg/g level.

### 3.3. Laboratory Materials

Careful selection of the labware materials, that contain or come in direct contact with ultrapure samples or reagents, is crucial because the trace element contents of different kinds of plastic are not satisfactory for ultra-trace analysis as they can introduce contaminants into the samples or absorb the analytes [10,11,17,56]. In our laboratory, we selected the appropriate materials from among the plastic materials used by C. Patterson and coworkers at the California Institute of Technology (CIT, Pasadena, CA, USA) for their Pb analysis [23], and by Boutron and coworkers at the Laboratoire de Glaciologie et Géophysique de l’Environnement (LGGE), Grenoble, France, for measuring ultralow trace elements in polar snow and ice [10,17]. At temperatures lower than ~60 °C or for diluted acid concentrations, LDPE bottles and containers (Nalgene Company, Rochester, NY, USA) were always preferred. For comparison, fluorinated ethylene propylene (FEP) or perfluoroalkoxy (PFA) Teflon was used for the storage of concentrated acids, heating of samples over ~60 °C, and field sampling equipment, such as cylindrical tubes and scrapers, requiring less fragile properties because of the below freezing temperatures in central Greenland. Polypropylene (PP) was used for the containers used to dispense ultrapure water during cleaning procedures within the laboratory, for the tips of the Eppendorf micropipettes, and for the tongs used to transfer all the items between the acid cleaning baths (see the next section). The only non-plastic material was the stainless steel (grade 316L) chisels used for decontaminating the ice core samples [18].

### 3.4. Cleaning Procedures

The cleaning procedures for LDPE bottles and other items generally followed those in the literature [10,17], such as immersing them in a series of acid cleaning baths at 30–35 °C. The acid baths with custom-made covers were made out of 20 L LDPE carboys from Nalgene (Rochester, NY, USA). Prior to acid-cleaning, the LDPE bottles and other items were initially degreased with Liquinox detergent (Alconox 1201) and rinsed with MQW whilst being held with custom-made PP tongs. They were then immersed for a week in each of four successive acid baths: the first (25% GR grade HNO_3_ diluted in MQW) at room temperature, followed by a series of three (25% Merck “Suprapur” grade HNO_3_ diluted in MQW for the second bath and 0.2% Fisher “Optima” grade ultrapure HNO_3_ diluted in MQW for the third and fourth) heated on hot plates at 30–35 °C (temperature of acid solution). Lower temperature heating, relative to the slightly higher temperatures (45–50 °C) employed by Boutron [10] and Hong et al. [17], reduced the risk of the acid bath corrosion during heating on hot plates. After final rinsing with MQW, they were filled with 0.1% Fisher “Optima” grade ultrapure HNO_3_ diluted in MQW and stored in sealed, acid-washed polyethylene bags until use. The FEP and PFA Teflon materials were first immersed in concentrated Merck “Suprapur” HNO_3_ at room temperature for at least a week, and subsequently cleaned, following the same procedure as that used for the LDPE items. PFA beakers used for pre-concentrating the samples by non-boiling evaporation were left immersed in the last acid bath until use. As they have remained immersed for over ~20 years [17], any contamination from the wall of these beakers is completely negligible when used for pre-concentrating the samples. Finally, stainless steel chisels were initially degreased with Liquinox detergent, immersed in concentrated Merck “Suprapur” HNO_3_ for several weeks at room temperature, and subsequently cleaned following the same procedure as that used for the LDPE items.

### 3.5. Sample Description and Dating

Snow pit samples were collected on June 26, 2009 at a site (77°26′N, 51°03′W, 2461 m a.s.l., mean annual snow accumulation rate of 22.5 g cm^−2^ yr^−1^) located ~3.5 km from the NEEM deep ice coring site in northwest central Greenland [57] (Figure S1). Stringent precautions were taken in the field to prevent the possibility of snow sample contamination. A 3.2-m deep snow pit was hand-dug by operators wearing full clean room garments and polyethylene gloves with acid-cleaned LDPE shovels. The upwind vertical wall of the pit was shaved away (~10 cm thick) before sampling, using acid-cleaned ultraclean Teflon scrapers. A continuous sequence of 70 samples was then collected from the surface down to the bottom by horizontally pushing acid-cleaned ultraclean cylindrical Teflon tubes (5 cm and 4.6 cm in outer and inner diameters, respectively, and 35 cm in length) into the snow, using an LDPE hammer. Snow samples were then transferred from the tubes into acid-cleaned ultraclean LDPE wide-mouthed 1 L bottles for storage [26,27,36,37]. The bottles were packed in double-sealed acid-cleaned LDPE bags and kept frozen at −20 °C until analysis. All the equipment used for field sampling was extensively cleaned as described above.

The NEEM snow pit samples had previously been dated by combining the depth profiles of stable water isotopes (δ^18^O) and ionic species (Na^+^, Ca^2+^, Cl^−^, SO_4_^2−^, and methanesulfonic acid (MSA)), they showed well-defined seasonal patterns of concentrations [26,27,36,37]. These strong seasonal patterns indicated that the NEEM snow samples covered a full 6-year period from spring 2003 to summer 2009 (Figure 1).

To assess the Holocene natural concentration levels of the trace elements investigated, we analyzed four sections selected from the 2540-m-long NEEM deep ice core [57]. The depths of these samples ranged from 1215.50 m to 1297.45 m, corresponding to ages of ~8056 and 9166 years BP, respectively, covering the early Holocene time interval. Each of the analyzed core sections (4 × 4 cm^2^ cross-section, 20 cm in length) was mechanically decontaminated before analysis, using sophisticated ultraclean procedures, as described in detail in Han et al. [18]. The contamination procedure involved the chiseling of successive veneer layers of the core in progression from the contaminated outside toward the center to obtain the contamination-free inner part of the core, using ultraclean stainless steel chisels inside a laminar flow class 100 clean bench in a cold room (−15 °C). The inner core was then divided into two pieces (each 10 cm long) and the upper piece was analyzed for this study. Each subsample covers a duration of ~0.5 year of snow accumulation.

### 3.6. Total Procedural Blank (TPB)

Contamination problems can occur at almost any stage of field sampling, storage, handling and analysis, causing a systematic error in ultra-trace analysis. However, it is essential to determine the TPB encountered during the complete procedure, from field sampling to analysis, in order to validate the reliable measurements of trace elements at or below the sub-pg/g, and as determining the magnitude of contamination introduced during individual stages is very difficult.

For the Greenland snow samples, we evaluated the TPB contribution via the following successive procedures. We first poured the ultrapure SDW, for which concentrations of selected trace elements were already known, into two acid-cleaned ultraclean cylindrical Teflon tubes used for Greenland snow sampling, left them for 2 h and then transferred the SDW to LDPE 1 L bottles in the Class 10 clean bench. The SDW was then pre-concentrated by non-boiling evaporation, acidified to 1% HNO_3_ with Fisher “Optima” grade ultrapure HNO_3_ and analyzed by ICP-SFMS. For the NEEM Holocene ice samples, the TPB was determined by carrying out the decontamination procedure, using four AICs prepared by freezing MQW inside 2 L PFA cylinders (Savillex Corporation, Eden Prairie, MN, USA) [18]. Prior to processing the AICs, they were cut with a band saw machine into square pillars to give a similar core section shape to that of the Greenland ice core sections to be decontaminated. Each cut AIC was mechanically decontaminated using exactly the same procedure as described in Han et al. [18]. Each layer and the final inner core, obtained after decontamination, were analyzed separately by ICP-SFMS.

### 3.7. Analytical Procedures

The snow pit samples were melted at room temperature in the original 1 L LDPE bottles inside a Class 10 clean bench and then immediately aliquoted for subsequent analyses of stable water isotopes, major ions, and trace elements. The aliquots for trace element analysis were acidified with Fisher “Optima” grade ultrapure HNO_3_ to make 1% solutions and then kept frozen until analysis. All analytical instruments were placed within a Class 10 laminar flow clean booth inside a Class 1000 clean room at KOPRI.

Stable water isotopes (δ^18^O) were analyzed using a Picarro L1102-I wavelength-scanned cavity ring-down spectrometer (WS-CRDS, Picarro Inc., Santa Clara, CA, USA). The isotopic measurements were converted to the Vienna Standard Mean Ocean Water (VSMOW2) and the Standard Light Antarctic Precipitation (SLAP2) scales by measuring standards of known isotopic composition [36]. Major ionic species were analyzed by ion chromatography with Dionex IonPac AS15 and CS12A columns for anions and cations, respectively, using Dionex ICS-2000 and ICS-2100 systems (Thermo Fisher Scientific Inc, Sunnyvale, CA, USA) [58].

Cd, U, and Zn concentrations were determined by ICP-SFMS (Element2, Thermo Fisher Scientific, Bremen, Germany) coupled with an APEX microflow nebulization desolvation system (APEX, HF, ESI, Omaha, NE, USA). Detection limits, defined as three times the standard deviation of 10 measurements of the blank (1% “Optima grade HNO_3_ diluted in SDW), were 0.009, 0.005, and 0.118 pg/g for Cd, U, and Zn, respectively (Table 1). Analyses of diluted solutions of certified riverine water reference materials, SLRS-6 (National Research Council, Ottawa, ON, Canada), exhibited very good recovery versus certified values (in pg/g): 8.7 ± 0.2 versus 6.3 ± 1.4 for Cd, 72 ± 0.4 versus 70 ± 3.4 for U, and 1873 ± 721 versus 1760 ± 120 for Zn.

## 4. Conclusions

This study has provided a basic overview of the ultraclean facilities and procedures that have been employed for determining trace elements at or below the pg/g concentration in Greenland snow and ice. Our study has demonstrated that incomplete ultraclean procedures from field sampling to laboratory analysis have caused unusually high concentrations, by orders of magnitude, relative to the concentration ranges that are considered reliable as reported values in Greenland snow and ice. This underscores the necessity of well-established ultraclean methods for ensuring reliable ultra-trace analysis. Our data reveal that the potential risk of contamination exposure for selected trace elements increases in the order of U < As ≤ Pb < Cd < Zn. The comprehensive ultraclean procedures employed in this study will facilitate the output of a wide range of reliable datasets from other investigations of ultralow trace element concentrations in sea water and Antarctic snow and ice.

## Figures and Tables

**Table 1:** Concentrations (in pg/g) of selected trace elements measured in ultrapure Milli-Q (MQW) and sub-boiling distilled (SDW) waters. TPB indicates the total procedural blank introduced due to the overall measurement procedure of snow samples and from the decontamination process of artificial ice cores (AICs) (see text).

Element	Detection Limit ^a^	MQW	SDW	TPB	
				Snow	AICs ^b^
As	0.292	0.029 (0.039) ^c^	0.045 (0.036) ^d^	0.003 (0.002) ^e^	0.108 (0.020) ^f^
Ba	0.056	0.077 (0.031)	0.036 (0.015)	0.005 (0.005)	<LOQ ^g^
Cd	0.009	0.008 (0.009)	0.003 (0.003)	0.0002 (0.0001)	0.016 (0.019)
Pb	0.026	0.286 (0.278)	0.150 (0.050)	0.005 (0.003)	0.061 (0.037)
U	0.005	0.001 (0.001)	0.001 (0.001)	0.0001 (0.0001)	0.009 (0.0003)
Zn	0.118	0.260 (0.171)	0.234 (0.122)	0.052 (0.020)	0.632 (0.198)

^a^ 3 times the standard deviation of 10 measurements of the blank. ^b^ TPB determined for the inner core. ^c–f^ In parentheses, standard deviation, *n* = 5, 9, 6 and 4, respectively. ^g^ Limit of quantitation.

**Table 2:** Statistical summary of mean values (expressed as mean ± SD) and ranges (min–max in parentheses) of elemental concentrations and crustal enrichment factors (EFs) in our snow and ice samples and comparison with published data obtained from Greenland snow and ice.

Site	Altitude (m a.s.l.)	Sampling Method	Period	Accumulation Rate (g H_2_O/cm^2^/yr)	Concentration (pg/g)					Ref.
					As	Cd	Pb	U	Zn	
NEEM (77°26′N, 51°03′W)	2461	Snow pit	2003–2009	22.5	2.63 ± 2.75 ^a^ (0.50–15.8)	0.97 ± 0.89 (0.07–5.57)	21.7 ± 22.7 ^b^ (2.7–97.3)	0.29 ± 0.37 (0.04–1.59)	46.4 ± 33.5 (6.97–156)	This study
**max/min**					32	80	36	40	22	[26]
**Enrichment factor**					54 ± 37 (12–163)	430 ± 376 (104–2196)	43 ± 20 (15–105)	3.5 ± 1.8 (1.4–13)	44 ± 37 (9–173)	[27]
Northwest and central Greenland	coast ~ 3500 m	Snow pit	spring 2012–2013, summer 2013–2014		22 ± 29	43 ± 50	200 ± 690	0.7 ± 1.1	4,120 ± 4,830	[28]
**Enrichment factor**					124 ± 101	5595 ± 1850	118 ± 147	2.8 ± 1.5	971 ± 156	
Summit (72°20′N, 38°45′W)	3270	Snow pit	1991–1995	23		1.15 ± 1.7 (0.1–11.6)	17.3 ± 17.7 (2.0–108)	1.75 ± 2.6 (0.2–15)	50.6 ± 49.4 (2–285)	[29]
**Enrichment factor**						326 ± 1066 (20–8731)	30 ± 59 (1.5–456)	15 ± 23 (1.1–132)	24 ± 32 (1.2–177)	
Summit (72°20′N, 38°45′W)	3270	Snow pit	1990–1992	23		0.67 (0.08–2.5)	15 (0.6–44)		51 (9–194)	[30]
Summit (72°20′N, 38°45′W)	3190	Snow pit	1981–1990	23		1.3 ± 0.91 (0.09–5.72)	43.6 ± 31.4 (4.37–158)			[31,32]
Summit (72°20′N, 38°45′W)	3270	Tube sampling	1989–1990	23		1.3 (0.3–3.7)	21.6 (3–50)		42 (2–144)	[33]
South Greenland (Dye 3) (65°11′N, 43°50′W)	2479	Tube sampling	1983–1984	50		0.74 (0.2–1.3)	28 (5–90)		27 (10–50)	[34]
NEEM (77°26′N, 51°03′W)	2461	Deep ice core	8266–9166 yr BP	22.5	2.15 ± 0.64 (1.44–2.69)	0.32 ± 0.11 (0.20–0.40)	1.60 ± 0.07 (1.55–1.67)	0.34 ± 0.14 (0.21–0.50)	33.8 ± 7.7 (27.1–42.2)	This study
**Enrichment factor**					19 ± 7 (14–27)	58 ± 27 (39–88)	1.8 ± 0.7 (1.0–2.2)	2.6 ± 1.8 (1.3–4.7)	12 ± 2.6 (8.7–13.7)	
		Deep ice core	8056 yr BP		1.47	2.03	24.4	0.06	2,880	
Summit (72°34′N, 37°37′W)	3238	GRIP deep ice core	8250–9310 yr BP	23		0.59 ± 0.10 (0.50–0.69)	0.90 ± 0.17 (0.79–1.10)		26 ± 6.08 (19–30)	[35]

^a^ from Lee et al. [26]. ^b^ from Kang et al. [27]

## Supplementary Materials

The following are available on line. Table S1: Trace element concentrations measured in the NEEM snow pit samples. Figure S1: Map of Greenland showing the location of the NEEM snow pit sampling and other sampling sites discussed in the text.
