# Supplementary material for: Reliable Ultra Trace Analysis of Cd, U and Zn Concentrations in Greenland Snow and Ice by Using Ultraclean Methods for Contamination Control
Source: Molecules. 2020 May 28;25(11):2519. doi: 10.3390/molecules25112519 (PMC7321221; doi:10.3390/molecules25112519)
Supplement: Supplementary file 1 [file molecules-25-02519-s001.pdf]

Supplementary Materials

Reliable ultra trace analysis of Cd, U and Zn concentrations in Greenland snow and ice by using ultraclean methods for contamination control

Changhee Han[1], Heejin Hwang[1], Jung-Ho Kang[1], Sang-Bum Hong[1], Yeongcheol Han[1], Khanghyun Lee[1], Soon Do Hur[1], Sungmin Hong[2]*

[1] Korea Polar Research Institute, 26 Songdomirae-ro, Yeonsu-gu, Incheon 21990, Korea

[2] Department of Ocean Sciences, Inha University, 100 Inha-ro, Michuhol-gu, Incheon 22212, Korea

[*] Correspondence: smhong@inha.ac.kr (S.H.); Tel: +82-32-860-7708

Summary

There are 6 pages in the Supplementary Material including 1 figure and 1 table.

Figure S1. Map of Greenland showing the NEEM snow pit and other sampling sites described in the text and Table 2. A total of 133 samples from 22 snow pits were collected by Lai et al. (2017) along a traverse route (solid lines) across northwest and central Greenland (see text). Background map from Google Earth Engine image.

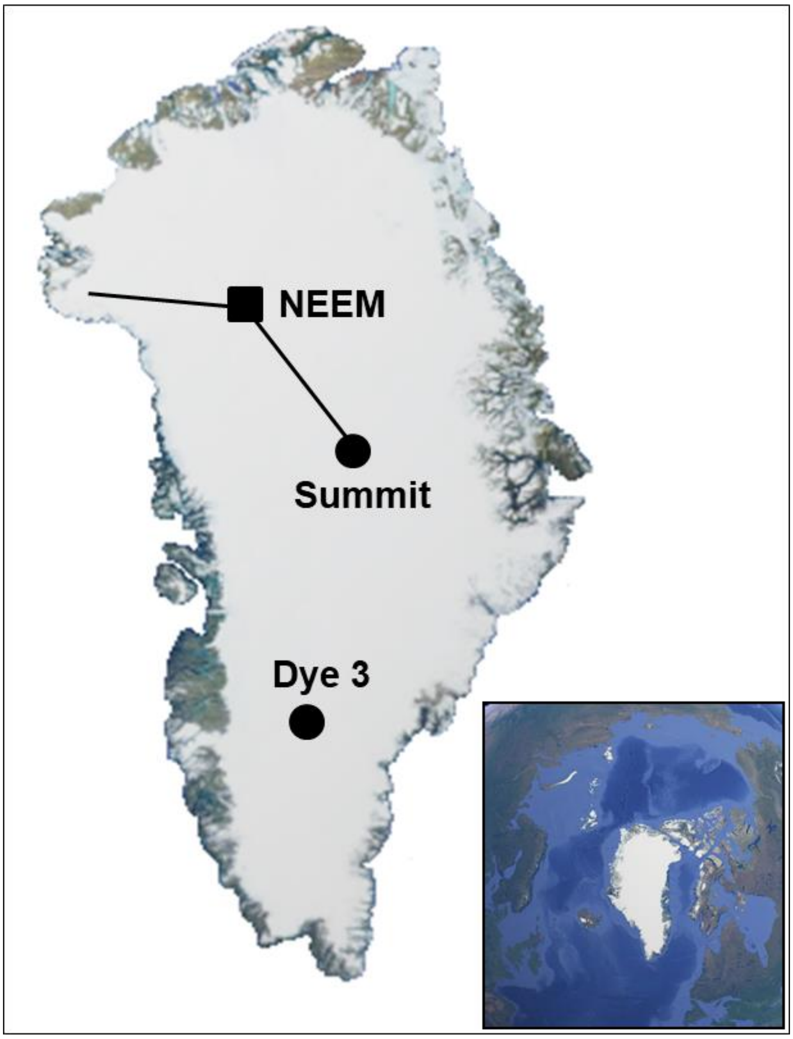

Table S1. Concentrations of Ba, Cd, U, and Zn and crustal enrichment factors for Cd, U, and Zn measured in a continuous series of 70 snow samples collected from a 3.2-m NEEM snow pit in northwest Greenland

Depth interval (cm)		Concentration (pg/g)				Enrichment factor		
Top	Bottom	Ba[a]	Cd	U	Zn	Cd	U	Zn
0	4.6	30.2	1.13	0.49	65.8	245	4.3	28
4.6	9.1	33.5	0.77	0.24	42.3	151	1.9	16
9.1	13.7	189	3.14	1.49	132	109	2.1	9
13.7	18.3	28.2	0.99	0.16	48.0	231	1.5	22
18.3	22.9	21.0	0.58	0.19	31.7	180	2.4	19
22.9	27.4	16.1	0.62	0.18	26.9	253	2.9	22
27.4	32.0	8.60	0.48	0.09	21.1	365	2.7	32
32.0	36.6	7.48	0.36	0.08	16.9	314	2.9	29
36.6	41.1	7.70	0.30	0.06	14.9	252	2.2	25
41.1	45.7	4.95	0.19	0.07	14.5	249	3.8	38
45.7	50.3	3.96	0.26	0.05	14.6	423	3.4	47
50.3	54.9	3.51	0.19	0.04	7.13	348	3.2	26
54.9	59.4	2.07	0.07	0.04	6.97	233	5.0	43
59.4	64.0	3.61	0.16	0.05	16.1	293	3.6	57
64.0	68.6	13.4	0.39	0.18	25.7	190	3.5	25
68.6	73.1	33.8	1.11	0.31	41.7	216	2.5	16
73.1	77.7	6.43	0.54	0.12	26.5	548	4.9	53
77.7	82.3	8.82	0.60	0.09	33.6	443	2.8	49
82.3	86.9	8.49	0.39	0.12	20.3	304	3.8	31
86.9	91.4	24.4	1.63	0.32	103	437	3.5	54
91.4	96.0	15.0	0.67	0.21	31.8	292	3.8	27
96.0	100.6	32.5	1.09	0.74	54.3	220	6.1	21
100.6	105.1	33.5	1.06	0.47	42.6	207	3.7	16
105.1	109.7	74.4	1.56	0.78	63.3	137	2.8	11
109.7	114.3	10.0	0.30	0.13	19.3	198	3.6	25
114.3	118.9	11.2	0.44	0.14	21.5	259	3.5	25
118.9	123.4	20.2	1.09	0.21	48.7	353	2.7	31
123.4	128.0	16.2	0.84	0.21	38.1	342	3.5	30

128.0	132.6	7.73	0.44	0.07	21.8	371	2.5	36
132.6	137.1	3.33	0.37	0.05	25.9	730	4.2	100
137.1	141.7	5.18	0.38	0.07	33.9	484	3.5	84
141.7	146.3	4.48	0.36	0.06	29.8	520	3.7	86
146.3	150.9	6.39	0.41	0.09	30.9	420	3.6	62
150.9	155.4	17.4	0.63	0.18	34.3	236	2.7	25
155.4	160.0	108	2.38	0.96	86.1	144	2.4	10
160.0	164.6	183	2.90	1.59	148	104	2.3	10
164.6	169.1	187	3.06	1.57	156	107	2.2	11
169.1	173.7	123	2.36	1.08	109	126	2.3	11
173.7	178.3	20.2	0.60	0.24	26.1	196	3.2	17
178.3	182.9	7.41	0.32	0.07	17.7	279	2.7	31
182.9	187.4	1.80	0.39	0.05	17.8	1410	7.6	127
187.4	192.0	4.22	0.67	0.06	36.6	1035	3.6	111
192.0	196.6	8.55	0.40	0.27	31.7	304	8.4	48
196.6	201.1	8.90	0.77	0.12	65.9	565	3.7	95
201.1	205.7	19.3	1.03	0.15	51.6	348	2.1	34
205.7	210.3	52.6	1.99	0.72	84.1	248	3.7	21
210.3	214.9	34.9	1.03	0.25	45.9	193	1.9	17
214.9	219.4	27.1	0.60	0.16	23.3	146	1.6	11
219.4	224.0	20.9	0.49	0.11	94.6	155	1.4	58
224.0	228.6	19.9	0.63	0.13	31.9	207	1.7	21
228.6	233.1	24.1	0.84	0.13	63.8	227	1.4	34
233.1	237.7	20.9	1.44	0.21	47.9	450	2.7	29
237.7	242.3	16.6	1.02	0.84	45.4	402	13.5	35
242.3	246.9	3.09	1.04	0.05	41.7	2196	4.2	173
246.9	251.4	2.48	0.40	0.04	20.9	1069	4.3	108
251.4	256.0	1.65	0.40	0.04	20.5	1576	6.3	160
256.0	260.6	12.3	1.92	0.10	104	1027	2.1	109
260.6	265.1	13.2	1.21	0.11	44.4	601	2.2	43
265.1	269.7	15.5	1.23	0.14	44.6	520	2.5	37
269.7	274.3	18.0	1.21	0.15	46.2	440	2.3	33
274.3	278.9	75.2	5.57	0.69	120	485	2.4	21

278.9	283.4	25.1	1.37	0.22	48.3	358	2.4	25
283.4	288.0	3.05	0.30	0.05	12.9	641	4.6	54
288.0	292.6	2.63	0.42	0.05	17.9	1049	4.8	87
292.6	297.1	4.82	0.52	0.05	29.3	709	2.7	78
297.1	301.7	1.92	0.35	0.04	18.2	1183	5.7	122
301.7	306.3	35.5	1.78	0.62	88.8	329	4.7	32
306.3	310.9	56.0	1.09	0.77	58.8	127	3.7	13
310.9	315.4	75.6	1.80	0.79	71.8	156	2.8	12
315.4	320.0	12.1	1.00	0.10	72.0	543	2.1	77
	Min	1.65	0.07	0.04	6.97	104	1.4	9
	Max	189	5.57	1.59	156	2196	13	173
	Mean	28.2	0.97	0.30	46.0	430	3.5	44
	SD	41.1	0.9	0.4	33.5	376	1.8	37

[a] From Kang et al. (2017)

References

Kang, J. –H.; Hwang, H.; Han, C.; Hur, S. D.; Kim, S. –J.; Hong, S. Pb concentrations and isotopic record preserved in northwest Greenland snow. *Chemosphere* **2017**, *187*, 294–301.

Lai, A. M., Shafer, M. M., Dibb, J. E., Polashenski, C. M., Schauer, J. J. Elements and inorganic ions as source tracers in recent Greenland snow. *Atmos. Environ*. **2017**, *164*, 205–215.
